# *Mycobacterium abscessus* subsp. *abscessus* Is Capable of Degrading *Pseudomonas aeruginosa* Quinolone Signals

**DOI:** 10.3389/fmicb.2017.00339

**Published:** 2017-03-02

**Authors:** Franziska S. Birmes, Timo Wolf, Thomas A. Kohl, Kai Rüger, Franz Bange, Jörn Kalinowski, Susanne Fetzner

**Affiliations:** ^1^Institute for Molecular Microbiology and Biotechnology, University of MünsterMünster, Germany; ^2^Center for Biotechnology (CeBiTec)Bielefeld, Germany; ^3^Research Center BorstelSülfeld, Germany; ^4^German Center for Infection ResearchBorstel, Germany; ^5^Institute for Medical Microbiology and Hospital Epidemiology, Hannover Medical SchoolHannover, Germany

**Keywords:** *Mycobacterium abscessus*, *Pseudomonas aeruginosa*, quorum sensing, quorum quenching, *Pseudomonas* quinolone signal, alkylquinolone degradation

## Abstract

*Pseudomonas aeruginosa* employs 2-heptyl-3-hydroxy-4(1*H*)-quinolone (the *Pseudomonas* quinolone signal, PQS) and 2-heptyl-4(1*H*)-quinolone (HHQ) as quorum sensing signal molecules, which contribute to a sophisticated regulatory network controlling the production of virulence factors and antimicrobials. We demonstrate that *Mycobacterium abscessus*^T^ and clinical *M. abscessus* isolates are capable of degrading these alkylquinolone signals. Genome sequences of 50 clinical *M. abscessus* isolates indicated the presence of *aqdRABC* genes, contributing to fast degradation of HHQ and PQS, in *M. abscessus* subsp. *abscessus* strains, but not in *M. abscessus* subsp. *bolletii* and *M. abscessus* subsp. *massiliense* isolates. A subset of 18 *M. a*. subsp. *abscessus* isolates contained the same five single nucleotide polymorphisms (SNPs) compared to the *aqd* region of the type strain. Interestingly, representatives of these isolates showed faster PQS degradation kinetics than the *M. abscessus* type strain. One of the SNPs is located in the predicted promoter region of the *aqdR* gene encoding a putative transcriptional regulator, and two others lead to a variant of the AqdC protein termed AqdC^II^, which differs in two amino acids from AqdC^I^ of the type strain. AqdC, the key enzyme of the degradation pathway, is a PQS dioxygenase catalyzing quinolone ring cleavage. While transcription of *aqdR* and *aqdC* is induced by PQS, transcript levels in a representative of the subset of 18 isolates were not significantly altered despite the detected SNP in the promoter region. However, purified recombinant AqdC^II^ and AqdC^I^ exhibit different kinetic properties, with approximate apparent *K*_m_ values for PQS of 14 μM and 37 μM, and *k*_cat_ values of 61 s^-1^ and 98 s^-1^, respectively, which may (at least in part) account for the observed differences in PQS degradation rates of the strains. In co-culture experiments of *P. aeruginosa* PAO1 and *M. abscessus*, strains harboring the *aqd* genes reduced the PQS levels, whereas mycobacteria lacking the *aqd* gene cluster even boosted PQS production. The results suggest that the presence and expression of the *aqd* genes in *M. abscessus* lead to a competitive advantage against *P. aeruginosa*.

## Introduction

*Pseudomonas aeruginosa* is an opportunistic pathogen regulating its virulence via a complex quorum sensing (QS) network. Besides *N*-acyl homoserine lactone-mediated *las* and *rhl* systems, it possesses an alkylquinolone (AQ) dependent QS system which uses PQS [the *Pseudomonas* quinolone signal, 2-heptyl-3-hydroxy-4(1*H*)-quinolone] and its biosynthetic precursor HHQ [2-heptyl-4(1*H*)-quinolone] as signal molecules (reviewed in Heeb et al., 2011; Huse and Whiteley, 2011). AQ signaling is involved in the regulation of a number of virulence factors such as the siderophore pyoverdine, the redox-active phenazine pigment pyocyanin, rhamnolipid biosurfactants, and the cytotoxic lectin and adhesin LecA (Deziel et al., 2005; Heeb et al., 2011; Huse and Whiteley, 2011).

Several QS-regulated exoproducts of *P. aeruginosa* do not only contribute to establishing infections of the host, but also act as antimicrobials. These may affect other species coexisting with *P. aeruginosa* in mixed microbial communities such as those infecting the lung of cystic fibrosis (CF) patients. Studies using laboratory co-cultures of *P. aeruginosa* with other bacteria support the hypothesis that QS-controlled exoproducts are important for competition. For example, hydrogen cyanide, rhamnolipids, and phenazines together promoted *P. aeruginosa* competitiveness in co-culture with *Burkholderia multivorans*, with hydrogen cyanide contributing the greatest effect (Smalley et al., 2015). Pyoverdine was found to contribute to growth inhibition of *B. cenocepacia* by *P. aeruginosa* (Costello et al., 2014), and another study showed that pyoverdine-mediated iron acquisition was responsible for growth suppression of *Corynebacterium glutamicum, Bacillus subtilis*, and *Staphylococcus aureus* by cell-free culture supernatants of *P. aeruginosa* (Lee et al., 2016). Thus, in polymicrobial communities, bacteria capable of quenching the production of *P. aeruginosa* antimicrobials by interference with its QS systems should have some advantage for survival and growth.

Besides acting as QS signals contributing to the regulation of *P. aeruginosa* exoproducts, PQS and HHQ have antagonistic effects on other microorganisms. PQS acts as an iron-trap (Bredenbruch et al., 2006; Diggle et al., 2007), HHQ exhibits bacteriostatic activity against several Gram-negative bacteria, and both PQS and HHQ repress motility in a range of bacteria (Reen et al., 2011). Moreover, the AQ biosynthetic pathway of *P. aeruginosa* besides 2-alkyl-4(1*H*)-quinolones and their 3-hydroxylated congeners also yields 2-alkyl-4-hydroxyquinoline-*N*-oxides (AQNOs), which act as antibiotics, inhibiting respiratory electron transfer at the cytochrome *bc*_1_ complex (Lightbown and Jackson, 1956; Cooley et al., 2005). 2-Heptyl-4-hydroxyquinoline-*N*-oxide (HQNO), along with siderophores produced by *P. aeruginosa*, drives *S. aureus* from aerobic respiration to fermentative metabolism. This is not only detrimental to *S. aureus* growth rates and fitness, but also results in the production of lactate that is preferentially utilized by *P. aeruginosa* (Filkins et al., 2015).

*Rhodococcus erythropolis* BG43, an isolate from soil, is the first bacterium described to be able to degrade HHQ and PQS (Müller et al., 2014). Two gene clusters *aqdA1B1C1* and *aqdRA2B2C2*, both inducible by PQS or a metabolite thereof, were shown to be involved in PQS and HHQ degradation (Müller et al., 2015). Considering their multiple biological functions, degradation of HHQ and PQS could serve for QS interference, or detoxification, or both. Interestingly, homologs to the entire *aqdRA2B2C2* cluster from *R. erythropolis* BG43 (locus tags XU06_RS29725 to XU06_RS29740; NZ_CP011296.1) are conserved in the genomes of some other actinobacteria, especially in representatives of the rapidly growing mycobacteria (RGM) such as strains of *M. fortuitum, M. mageritense*, and *M. abscessus* (locus tags MAB_0300c to MAB_0303; NC_010397.1; Müller et al., 2015). *M. abscessus*, one of the most pathogenic and antibiotic-resistant RGM, is considered an emerging pathogen, causing a pseudotuberculosis lung disease to which patients with CF are particularly susceptible (Griffith et al., 2007; Roux et al., 2009). A recent study revealed that dominant clones of *M. abscessus*, which emerged a few decades ago and show increased virulence, have spread globally, emerging as a major threat to individuals with CF (Bryant et al., 2016).

The identification of genes potentially coding for an AQ conversion pathway in the genomes of *M. abscessus* raises the question of whether the strains are indeed able to transform AQ compounds. In this study, we analyzed the degradation of HHQ and PQS by *M. abscessus*^T^ DSM 44196 and by *M. abscessus* isolates from CF patients. Having identified three groups of strains that differ in their kinetics of PQS degradation and in the presence and type of *aqd* genes, we determined their effect on PQS levels in co-cultures with *P. aeruginosa*. To find out whether the differences in PQS degradation kinetics observed for the two groups of clinical isolates harboring *aqd* genes are due to distinct catalytic properties of their PQS dioxygenases, or due to differences in gene expression, we compared the kinetic properties of the purified enzymes and determined *aqd* transcript levels.

## Materials and Methods

### *Mycobacterium abscessus* Strains

The nomenclature of *Mycobacterium abscessus* is complicated by the non-uniform use in the literature of species and subspecies designations. Currently, two subspecies are recognized: *M. abscessus* subsp. *abscessus*, and *M. abscessus* subsp. *bolletii* which unites the previous subspecies *massiliense* and *bolletii* (Leao et al., 2011). However, for the sake of clarity and because recent publications support the previous classification (Cho et al., 2013; Tan et al., 2015; Bryant et al., 2016; Tortoli et al., 2016), we use the three-subspecies designations. The *M. abscessus* isolates from CF patients used in this study were characterized previously (Rüger et al., 2014; Kehrmann et al., 2016). The type strain of *M. abscessus* subsp. *abscessus* (DSM 44196) was obtained from DSMZ, Braunschweig, Germany.

### Chemicals

*Pseudomonas* quinolone signal and HHQ were purchased from Sigma Aldrich and dissolved in methanol.

### Genome Sequencing

Sequencing libraries were constructed from extracted genomic DNA with the Nextera XT kit (Illumina) and sequenced on the Illumina MiSeq instrument in a 2 × 300 bp paired end run or in a HiSeq 2 × 150 bp paired end Rapid Run.

### Reverse Transcription PCR

For isolation of RNA, *M. abscessus*^T^ (DSM 44196) was grown in DSM219 medium. To possibly induce the expression of *aqd* genes, 20 μM PQS was added 2 h before harvesting the cells by centrifugation. Cells were frozen in liquid nitrogen and stored at –80°C. Cells were then resuspended in TE buffer (10 mM Tris, 1 mM EDTA, pH 8.0) and disrupted using the Mikro-Dismembrator S (Sartorius, 3000 rpm, 2 min) after addition of glass beads (150–212 μm diameter) to the cell suspension. Subsequently, RNA purification was performed with the innuPREP RNA Mini kit (Analytik Jena) according to the manufacturer’s instructions. RNA concentration was determined using the Nanophotometer N60 (IMPLEN). Removal of DNA contamination was executed with DNAse I (Thermo Scientific) at 37°C and checked via PCR (GoTaq Polymerase, Promega) after renewed RNA purification. For analysis of operon structures, cDNA synthesis was carried out using the RevertAid H Minus First Strand cDNA Synthesis kit (Thermo Scientific) according to the manufacturer’s instructions. Primers were designed to amplify 500 bps of regions spanning *aqdAB* and *aqdBC*.

For relative mRNA quantification of single genes, RT-qPCR was used. Primers were designed to amplify 75–150 bps of the analyzed genes (sequences shown in **Table 1**). All measurements were performed in a LightCycler 96 System (Roche) with a SensiFast SYBR No-Rox One-Step Kit (Bioline, London, UK) and 96 well lightcycler plates (Sarstedt) as reaction vessels, sealed with qPCR seals (Sarstedt). The concentrations of all template RNA samples were adjusted to 200 ng/μL for normalization on total RNA. One microlitre of the RNA samples was used as template and mixed with 19 μL master mix containing 1 μL of specific primers (10 μM each), 0.2 μL reverse transcriptase, 0.4 μL RNase inhibitor, 10 μL reaction mix and 7.4 μL 5 M glycine betaine. All measurements were carried out with a minimum of three biological replicates in two technical duplicates each. For each primer pair two negative controls with 1 μL H_2_O as template were included. Reverse transcription was carried out at 45°C for 20 min, followed by 2 min polymerase activation at 95°C, a three step amplification (95°C 5 s, 60°C 10 s, 72°C 10 s, 60 cycles) and a melting profile analysis. Evaluation of control measurements and analysis of the melting curves as well as *C*_q_ calculation was carried out with the LightCycler 96 V1.1 software. The relative transcript amount was normalized on total RNA (200 ng) and calculated as 2^-ΔCq^ where Δ*C*_q_ corresponds to the difference of the mean *C*_q_ values.

**Table 1:** Primer sequences used for RT-PCRs.

Name	Sequence (5′ → 3′)	Application
regionAB-for	TGCTATTCGGGGATGAGGC	Determination of cluster organization
regionAB-rev	CATATGCATCGTCAAGCCCC	
regionBC-for	CGTATCAGAGAGCGCCGAT	
regionBC-rev	CGCCATCTCGTCAATACCGA	

*aqdC*-for	CGATCGGAATCTAGTTGGCG	RT-qPCR of *aqdC*
*aqdC*-rev	GAAACTGTCCACCTCAAGCG	

*aqdR*-for	TCGACCGAGAAGAAACCACA	RT-qPCR of *aqdR*
*aqdR*-rev	ATCCGTGTTTGTTCGATGCC	

16S-for	CAGGGCTTCACACATGCTAC	RT-qPCR internal control gene
16S-rev	AGACCCCAATCCGAACTGAG	

### Expression of *aqd* Genes in *E. coli* for Biotransformation Experiments

The *aqdB* and *aqdC* genes were amplified by PCR from a colony of *M. abscessus*^T^ (DSM 44196). Both AqdB and AqdC were produced as His_8_-MBP fusion proteins in recombinant *E. coli* strains, obtained by restriction-free cloning of the corresponding genes into the pET28b(+) expression vector (van den Ent and Löwe, 2006). *E. coli* Rosetta(DE3) was transformed with pET28b(+)::*his8*-*mbp-aqdB* due to many rare codons in the *aqdB* sequence. *E. coli* BL21(DE3) was transformed with pET28b(+)::*his8*-*mbp-aqdC*.

### Growth Conditions and Biotransformation Assays

*Mycobacterium* strains were cultivated in DSM219 medium, and recombinant *E. coli* BL21(DE3) and Rosetta(DE3) strains were grown in LB medium supplemented with 50 μg/mL kanamycin, at 37°C. For AQ biotransformation by mycobacteria, cells from pre-cultures were suspended at an optical density at 600 nm of 3.5 in fresh DSM219 medium, supplemented with 20 μM of HHQ or PQS, and incubated at 37°C. AQs were extracted at different time points as described previously (Müller et al., 2014). Samples of extracted cell suspensions were solubilized in methanol and analyzed via HPLC. For biotransformations of AQs by recombinant *E. coli* strains harboring pET28b(+)::*his8*-*mbp*-*aqdB* or pET28b(+)::*his8*-*mbp*-*aqdC*, cells were grown overnight at 30°C in the presence of 0.1 mM IPTG, harvested by centrifugation, and resuspended in fresh LB medium with 0.1 mM IPTG, adjusting an OD_600nm_ of 3.5. Biotransformation assays were performed as described above.

### Preparation of *Mycobacterium* Cell Extracts

Cell extracts were prepared from *M. abscessus*^T^ (DSM 44196) grown in DSM219 medium. To possibly induce the expression of AQ-converting enzymes, 20 μM PQS was added 2 h before harvesting the cells by centrifugation. Cells resuspended in 50 mM potassium phosphate buffer pH 7.5 were disrupted by sonication, and cell debris was removed by centrifugation (20.000 × *g*, 45 min, 4°C). The supernatant (crude extract) was desalted using Zeba^TM^ Spin Desalting columns (10 K molecular weight cut-off, Thermo Scientific). Total protein amount in cell extract supernatants was determined using the Bradford method as modified by Zor and Selinger (1996).

### Purification of AqdC Proteins

The sequence of *aqdC* of *M. abscessus*^T^ was optimized for codon usage of *E. coli* using OPTIMIZER (Puigbò et al., 2007) and synthesized by MWG Eurofins. For the purification of AqdC proteins, codon-optimized synthetic genes were cloned in pET28b(+) using restriction-free cloning (van den Ent and Löwe, 2006). The sequence coding for TEV protease cleavage site was introduced between the coding sequences for his_8_-tag and AqdC protein. In the following, AqdC^I^ refers to the protein of the type strain, and the protein which differs from AqdC^I^ by the two amino acid substitutions R129P and A133T is termed AqdC^II^. *E. coli* BL21(DE3) harboring pET28b(+)::*his8-aqdC*^I^ or pET28b(+)::*his8-aqdC*^II^ were grown at 37°C in Terrific Broth. At an OD_600nm_ of 1.0, cultures were supplemented with 0.2 mM IPTG and incubated at 16°C overnight (for approximately 16 h). Cells harvested by centrifugation were resuspended in washing buffer (300 mM NaCl, 20 mM Tris and 10 mM imidazole, pH 8.0), disrupted by sonication, and the AqdC proteins were purified by Ni-NTA affinity chromatography and stored in buffer containing 20 mM Tris, 10% (v/v) glycerol (pH 8.0) at -80°C.

### Enzyme Assay

The catalytic activity of AqdC proteins was determined spectrophotometrically at 30°C by measuring PQS consumption at 337 nm. The assays contained 20 μM PQS in assay buffer (50 mM Tris, 2 mM EDTA, 10% PEG 1500, 4% (v/v) DMSO, pH 8.0). The extinction coefficient of PQS in assay buffer is 10169 M^-1^ cm^-1^ at 337 nm. Apparent steady-state kinetic constants of AqdC proteins (two biological replicates with three technical replicates each) were estimated by fitting the initial velocities measured at different substrate concentrations with the Michaelis Menten equation.

### Cocultivation of *P. aeruginosa* and *M. abscessus* Clinical Isolates

Overnight cultures of *P. aeruginosa* PAO1 and *M. abscessus* strains were used as inocula for co-cultivation experiments in 10% LB medium. To account for differences in growth rates (generation times of *M. abscessus* and *P. aeruginosa* in 10% LB are 38.5 and 16.5 h, respectively), *P. aeruginosa* was adjusted to an initial OD_600nm_ of 0.05, and the mycobacterial strain to an OD_600nm_ of 0.15, as described by Costa et al. (2015). Cells were incubated at 37°C under vigorous shaking. PQS was extracted after 8 and 24 h as described previously (Müller et al., 2014) and quantified by HPLC analysis. Colony forming units (CFUs) of *P. aeruginosa* PAO1 were determined by dropping 10 μL of a diluted culture onto LB agar and counting colonies after incubation at 37°C overnight. *M. abscessus* formed colonies only after 36 to 48 h, so selective medium was not necessary.

### HPLC Analysis

For the identification and quantification of PQS and other AQs, compounds were separated on a 250 × 4 mm Eurospher II RP-18 column using a Hitachi EZchrom Elite HPLC system with diode array detector model 2450, or an Agilent 1100 series system with diode array detector model G1315B. Methanol with 0.1% (w/v) citric acid and 0.1% (w/v) citric acid in water were used as solvents. Separation of PQS and HHQ was carried out via a linear gradient from 80 to 100% methanol (v/v) over 20 min at a flow rate of 0.5 mL min^-1^.

## Results

### AQ Degradation in *M. abscessus*^T^

**FIGURE 1:**
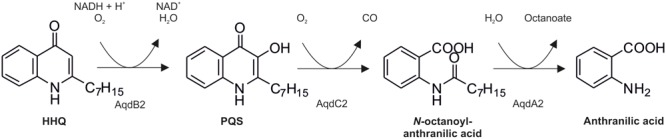
**Pathway of 2-heptyl-4(1*H*)-quinolone (HHQ) and *Pseudomonas* quinolone signal (PQS) conversion in *R. erythropolis* BG43 (Müller et al., 2015).** AqdB2, HHQ 3-monooxygenase; AqdC2, PQS 2,4-dioxygenase; AqdA2, *N*-octanoylanthranilate amide hydrolase.

**FIGURE 2:**
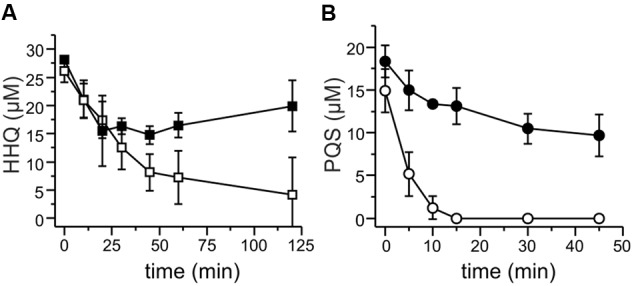
**HHQ and PQS conversion by cell extracts of *M. abscessus*^T^.** Extracts were obtained from cells grown in DSM219 medium (filled symbols), and from cells grown in the same medium but supplemented with PQS 2 h prior to harvesting (open symbols). Extracts obtained by sonication of cell suspensions were centrifuged, the resulting supernatants were desalted, set to a protein concentration of 1 mg/mL in 50 mM potassium phosphate buffer pH 7.5, and incubated with 20 μM HHQ and 500 μM NADH **(A)**, or with 20 μM PQS **(B)**. Samples were withdrawn at the given time points, extracted, and analyzed by HPLC. Means ± SD of three biological replicates are shown.

**FIGURE 3:**
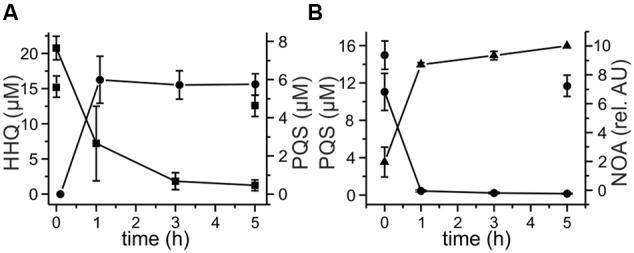
**Degradation of 20 μM HHQ (A)** and PQS **(B)** by recombinant *E. coli* strains. Cells suspended in LB medium were induced with 0.5 mM IPTG and incubated at 30°C overnight. Then they were supplemented with 20 μM HHQ or PQS and incubated at 30°C. Extracts of culture samples were analyzed by HPLC. **(A)** HHQ conversion by *E. coli* Rosetta pET28b::*mbp-aqdB*, squares: HHQ, circles: PQS. **(B)** PQS conversion by *E. coli* BL21 pET28b::*mbp-aqdC*; circles: PQS, triangles: *N*-octanoylanthranilic acid (NOA). *E. coli* Rosetta and *E. coli* BL21 did not convert HHQ (discrete squares in **A**) and PQS (discrete circles in **B**).

The presence of an *aqdRABC* gene cluster in the genome of *M. abscessus*^T^ suggested that it might degrade HHQ and PQS via reactions analogous to those identified in *R. erythropolis* BG43 (**Figure 1**). The *aqdB2* gene of *R. erythropolis* BG43 codes for an NADH-dependent HHQ monooxygenase, whereas the gene product of *aqdC2* is a PQS-cleaving dioxygenase which requires O_2_ as only co-substrate (Müller et al., 2015). AqdB and AqdC activity was present in cell extract supernatant suggesting cytoplasmic localization. Desalted cell extract supernatants of *M. abscessus*^T^ supplemented with NADH transformed HHQ (**Figure 2A**), and HHQ consumption was accompanied by transient formation of 2.0 ± 0.3 μM PQS, as identified by HPLC. Interestingly, extracts from cells pre-incubated with PQS converted HHQ and especially PQS faster than extracts from non-induced cells (**Figure 2**). To analyze whether transformation of HHQ and PQS is indeed catalyzed by the mycobacterial AqdB and AqdC protein, respectively, recombinant *E. coli* strains expressing *aqdB* or *aqdC* of *M. abscessus*^T^ were constructed and tested for AQ biotransformation. *E. coli* cells producing the His_8_-MBP-AqdB protein converted HHQ to PQS, and *E. coli* cells expressing the *M. abscessus*^T^ His_8_-MBP-AqdC fusion protein were able to cleave PQS to *N*-octanoylanthranilic acid, as identified by HPLC and comparison of UV/Vis spectra and fluorescent properties with reference compounds (**Figure 3**).

Taken together, the observations strongly support the hypothesis that the mycobacterial *aqdRABC* gene cluster (locus tags MAB_0300c to MAB_0303 in NC_010397.1) codes for an inducible AQ degradation pathway which proceeds analogous to that identified in *R. erythropolis* BG43 (**Figure 1**). *N*-Octanoylanthranilic acid formed by the PQS dioxygenase AqdC presumably is hydrolyzed by AqdA, a member of the carboxylesterase type B family, to anthranilic acid and octanoate, which both can be channeled into the central metabolism. The *aqdR* gene codes for a putative transcriptional regulator of the TetR family.

### Presence of *aqd* Genes in *M. abscessus* Strains Isolated from CF Patients

The genomes of *M. abscessus* strains, previously isolated from respiratory samples of CF patients (Rüger et al., 2014), were analyzed for the presence and sequence of the *aqd* gene cluster with BLASTN analyses (Altschul et al., 1997). Among these 50 strains, 22 (44%) lack the *aqd* gene cluster. Interestingly, absence of the *aqd* genes correlates with the assignment of the strains to the subspecies *bolletii* (3 strains) and *massiliense* (19 strains), whereas all 28 strains of the subspecies *abscessus* harbor the *aqd* gene cluster. In all cases where two isolates were obtained from the same patient, the nucleotide sequences of the *aqd* gene clusters did not differ between the first and second isolate.

The nucleotide sequences of the *aqdRABC* genes of strains P5a, P5n, P23a, P23n, P28a, and P28n are identical to those of *M. abscessus* subsp. *abscessus*^T^ (DSM 44196, ATCC 19977). Compared to the *aqd* gene region of the type strain, those of strains P13, P24 and another 16 isolates contain the same five single nucleotide variations (SNPs). Two of these SNPs within the protein coding regions of AqdA (triplet encoding L173) and AqdC (triplet encoding I187) are silent, and one is a transition within the predicted promoter region of *aqdR*. The two other SNPs lead to differences in the amino acids at position 129 and 133 of the predicted PQS dioxygenase AqdC (**Table 2** and Supplementary Figure 1). Two other *aqd* gene modifications observed in the genome of individual strains lead to single amino acid deviations in AqdA (G394S) and AqdB (G15S), respectively (**Table 2** and Supplementary Figure 1). However, with respect to AqdC, the key enzyme in PQS degradation, the strains can be divided into two groups: Nine isolates produce the same protein as the type strain (AqdC^I^), and 18 strains form a protein (AqdC^II^) which differs in two amino acids (**Table 2**). Supplementary Figure 1 shows the translated nucleotide sequence of the *aqdRABC* region and indicates the changes in nucleotide and amino acid sequences.

**Table 2:** Prediction of *aqd* gene products from the genomes of clinical isolates of *M. abscessus* (Rüger et al., 2014; Kehrmann et al., 2016).

Strain (month/year of isolation)	Subspecies	Aqd proteins compared to those of *M. abscessus*^T^
**P5a** (5/2001), **P5n** (11/2010), P23a, P23n, P28a, P28n	*abscessus*	Identical
P2a_s, P2a_r, P2n	*abscessus*	AqdB_G15S
**P13, P24** P1a, P1n, P14a, P14n, P15, P25, P26, P30, P31_s, P31_r, P33, P36a, P36n, P38, P39, P41	*abscessus*	AqdC_R129P_A133T
P10	*abscessus*	AqdA_G394S, AqdC_R129P_A133T
**P4a** (12/2002), **P4n** (3/2007) **P6a** (8/2007), **P6n** (10/2011), **P29, P32**, P3a, P3n, P7a, P7n, P8a, P8n, P11, P16, P17_r, P17_s, P18, P19, P35	*massiliense*	Absent
P37a, P37n, P40	*bolletii*	Absent

*Strains designated with the same number were isolated from the same patient. Designations set in bold indicate strains analyzed for HHQ and PQS biotransformation (for pairs of strains from the same patient the date of isolation is given in parentheses).*

### AQ Degradation by Clinical *M. abscessus* Isolates

**FIGURE 4:**
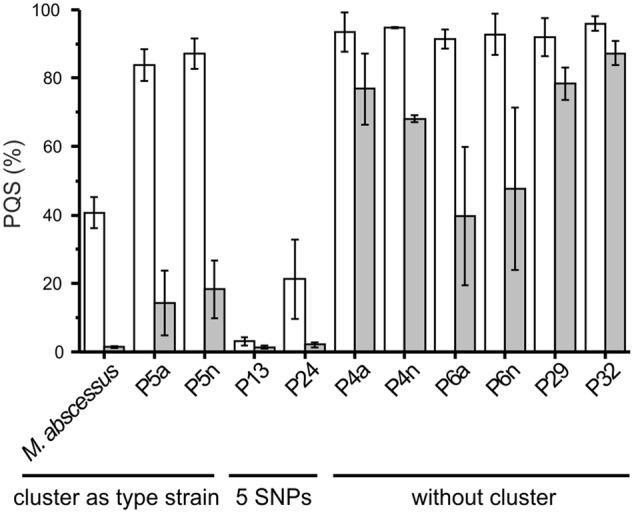
***Pseudomonas* quinolone signal consumption by the type strain and clinical isolates of *Mycobacterium abscessus*.** Cells suspended at an OD_600 nm_ of 3.5 in DSM219 medium were supplemented with 20 μM PQS and incubated at 37°C. Extracts of culture samples withdrawn after 0, 4 and 24 h were analyzed by HPLC. The PQS concentrations in samples taken at *t* = 0 h were set as 100%. Means ± SE of three independent biological replicates are shown. Decrease in PQS concentrations in sterile DSM219 medium due to abiotic oxidation was not observed within 24 h.

Ten of the clinical *M. abscessus* isolates (**Table 2**) were analyzed for their ability to degrade PQS and HHQ. Interestingly, members of the subset of 18 strains carrying the *aqd* gene cluster with the five SNPs (as compared to that of *M. abscessus*^T^) appeared to be the more potent PQS degraders. Surprisingly, even those strains that do not harbor *aqd* genes reduced the amount of PQS to different extents within 24 h (**Figure 4**).

**FIGURE 5:**
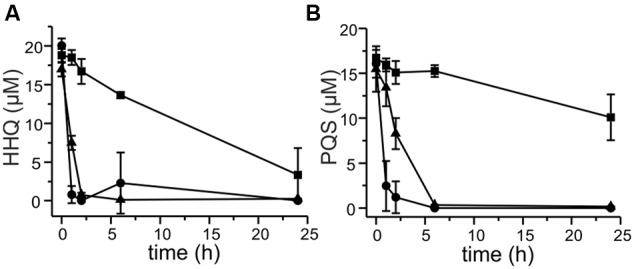
**Degradation of 20 μM HHQ (A)** and PQS **(B)** by *M. abscessus* strains. Cells suspended at an OD_600 nm_ of 3.5 in DSM219 medium were supplemented with 20 μM HHQ or PQS, and incubated at 37°C. Extracts of culture samples were analyzed by HPLC. Squares: *M. abscessus* P4a, circles: *M. abscessus* P13, triangles: *M. abscessus* P5a. Means ± SD of three biological replicates are shown.

We selected a representative of each of the groups of strains – strain P5a with an *aqd* gene cluster identical to that of *M. a.* subsp. *abscessus*^T^, strain P13 with the five SNPs in the *aqd* region, and strain P4a lacking *aqd* genes – to determine the time course of HHQ and PQS conversion by cell suspensions. HHQ consumption by strains P5a and P13 followed similar kinetics. However, as already suggested by the preliminary data shown in **Figure 4**, strain P13 converted PQS faster than strain P5a, and strain P4a lacking the gene cluster very slowly converted HHQ as well as PQS (**Figures 5A,B**).

Strains without the *aqd*-cluster which slowly consume PQS and HHQ must use alternative enzymes to modify or even degrade these AQs. HPLC analyses of culture extracts revealed transient formation of 4.9 ± 3.9 μM PQS after 2 h of incubation with 20 μM HHQ, besides an arsenal of other metabolites. Thus, it appears that other monooxygenases besides AqdB can mediate HHQ hydroxylation to PQS. Due to the complexity of metabolites formed, incomplete peak separations, and the low concentrations of intermediates (expected to be produced by the cultures at the nM range), elucidation of their structures will require the establishment of improved extraction and separation protocols, as well as considerable upscaling, to enable NMR analyses for structural identification. However, because the UV spectra of many of the peaks of the HPLC elution profile resemble that of PQS, we assume that the corresponding metabolites are quinolones, with modifications introduced mainly to the alkyl chain.

### Transcription of *aqd* Genes in Strains P13 and P5a

To analyze whether the *aqdABC* genes are co-transcribed, RT-PCR was performed with primer pairs addressing adjacent gene transcripts (for primer sequences see **Table 1**). Formation of PCR products, as verified by gel electrophoresis (not shown), indicate that *aqdAB* and *aqdBC* are co-transcribed, suggesting organization of the *aqdABC* genes in an operon.

**FIGURE 6:**
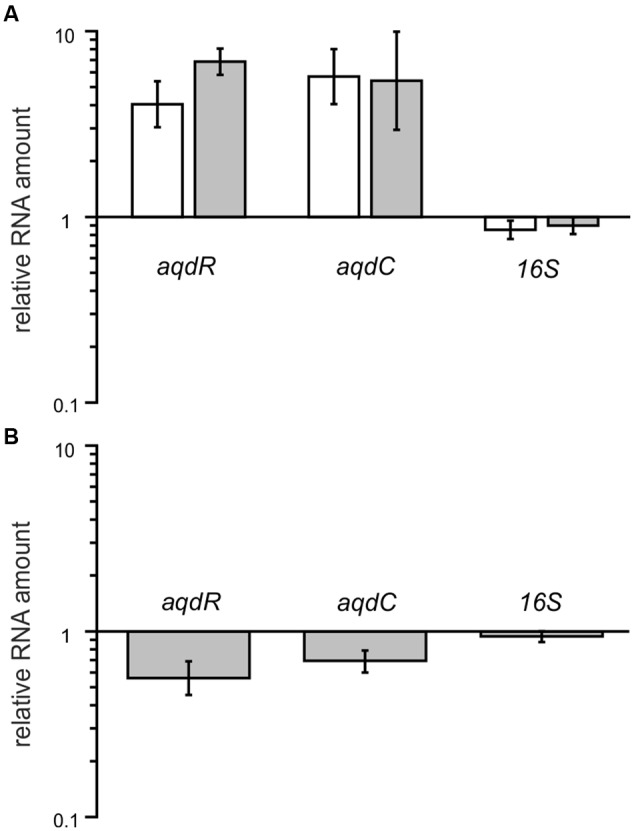
**Relative transcript levels of *aqdR, aqdC*, and 16S rRNA gene as a control in strains P5a and P13. (A)** Fold change in gene transcription due to PQS induction. White bars: strain P5a, gray bars: strain P13. Transcript amounts in non-induced cells were set as 1. **(B)** Transcript amounts of induced P13 cells normalized to levels of induced P5a cells. Means ± SD of at least three biological replicates are shown.

Cells induced with 20 μM PQS have higher transcript levels of *aqdR* (fold change between 4.0 and 6.8), indicating autoregulation of *aqdR* transcription. Transcript levels of *aqdC* were also increased when the cells were induced with PQS (fold change between 5.4 and 5.7) (**Figure 6A**). In strains harboring the *aqd* genes with five SNPs compared to the *aqd* region of the type strain, the transition within the predicted promoter region of *aqdR* changes the inverted repeat sequence TTGTCGCATCGACAA to TCGTCGCATCGACAA. To determine whether the SNP results in different expression levels, transcript levels of *aqdR* and *aqdC* were compared for strains P5a and P13. RT-qPCR analyses revealed only slight reduction of transcription amounts in strain P13 (**Figure 6B**). Thus, it seems unlikely that differential expression of the *aqdC* gene coding for the key enzyme, a PQS dioxygenase, accounts for the observed differences in PQS degradation rates (**Figure 5**).

### Catalytic Activity of AqdC Proteins

To analyze the possibility that the AqdC^I^ and AqdC^II^ proteins differ in their catalytic efficiency, recombinant proteins were purified to electrophoretic homogeneity, and their steady-state kinetic parameters were determined. Under the conditions of the assay, a specific activity of 53.2 U mg^-1^, an apparent *k*_cat_ of 97.8 ± 19.1 s^-1^ and an apparent *K*_m_ value for PQS of 37.2 ± 9.8 μM were observed for AqdC^I^, the PQS dioxygenase form of the *M. a*. subsp *abscessus* type strain and the group of strains represented by the isolate P5a. For AqdC^II^, the enzyme of the group of isolates represented by strain P13,a specific activity of 50.5 U mg^-1^, an apparent *k*_cat_ of 61.0 ± 4.3 s^-1^ and apparent *K*_m_ value of 13.6 ± 1.6 μM were determined.

### PQS Concentrations in Co-cultures of *P. aeruginosa* and *M. abscessus*

**FIGURE 7:**
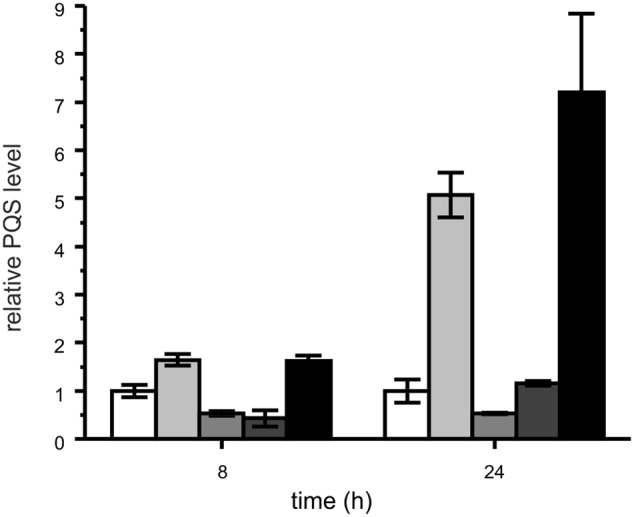
**Relative PQS levels in cultures of *P. aeruginosa* PAO1 (white bars) and co-cultures of *P. aeruginosa* PAO1 with *M. abscessus* P4a (light gray), *M. abscessus* P5a (gray), *M. abscessus* P13 (dark gray), and autoclaved *M. abscessus* P13 (black), incubated under shaking at 37°C.** Extracts of cultures were analyzed by HPLC. PQS concentrations in *P. aeruginosa* PAO1 cultures were set as 1 (8 h: 1.79 μM; 24 h: 0.76 μM PQS). Means ± SD of three biological replicates are shown. CFUs of *P. aeruginosa* PAO1 after 24 h of incubation: PAO1 culture: 1.81 × 10^11^; PAO1 – P13 co-culture: 1.21 × 10^11^; PAO1 cultured with autoclaved P13: 1.32 × 10^11^.

Representatives of each *M. abscessus* group were co-cultivated with *P. aeruginosa* PAO1 to test whether PQS produced by *P. aeruginosa*, which is packaged into membrane vesicles for trafficking between cells (Mashburn-Warren et al., 2008), is amenable to degradation by the mycobacteria. Indeed, the clinical isolates P5a and P13, which degraded synthetic PQS, also were able to reduce the PQS concentration in co-culture with *P. aeruginosa* PAO1 (**Figure 7**). Most interestingly, however, the PQS concentration increased significantly when *P. aeruginosa* PAO1 was cultivated with strain P4a, a representative of the group lacking the *aqd* genes. Compared with the *P. aeruginosa* PAO1 cultures, the amount of PQS in *P. aeruginosa* PAO1 – *M. abscessus* P4a co-cultures was fivefold higher after 24 h of cultivation. *P. aeruginosa* PAO1 cultivated with dead (autoclaved) *M. abscessus* strain P13 cells produced increased amounts of PQS as well. After 24 h of incubation, the PQS concentration was almost eightfold higher than in the PAO1 solo cultures. CFUs of *P. aeruginosa* PAO1 cultures were similar to those of PAO1 in co-culture with *M. abscessus* strain P13, or PAO1 cultivated in the presence of autoclaved *M. abscessus* P13 cells. Therefore, the increase in PQS production especially in presence of autoclaved cells is not due to increased population density of *P. aeruginosa*. The boosting of PQS production may rely on the recognition of cell components by PAO1. It makes the reduced levels of PQS in PAO1-P5a and PAO1-P13 co-cultures even more remarkable.

## Discussion

*Pseudomonas aeruginosa* often dominates the microbiome of the lungs of adult CF patients, however, the CF lung is usually colonized with multiple pathogens. CF-related lung disease also is a risk factor for chronic pulmonary infection with RGM, and RGM are actually detected with increasing prevalence in the CF population. Especially *M. abscessus* is considered an emerging threat to individuals with CF (Bar-On et al., 2015; Bryant et al., 2016).

Alkylquinolones produced by *P. aeruginosa*, acting as QS signals and antimicrobials, have been detected in the sputum of CF patients (Collier et al., 2002; Barr et al., 2015). Thus, *M. abscessus*, when co-colonizing the CF lung, may well encounter these secondary metabolites. In this study, we demonstrate that *M. abscessus* is capable of degrading the *P. aeruginosa* signal molecules HHQ and PQS. Interestingly, clinical *M. abscessus* strains isolated from patients with CF showed different kinetics in PQS degradation, correlating with the presence and type of *aqdRABC* genes coding for an inducible HHQ and PQS degradation pathway. Differences in transcript levels, possibly related with the SNP in the promoter of the *aqdR* gene, differences in the kinetic properties of the PQS dioxygenases AqdC^I^ and AqdC^II^, and additional factors such as mRNA or protein stability might influence the degradation rates. While RT-qPCRs showed no significant differences in expression levels of the *aqdR* and *aqdC* genes of strains P13 and P5a, the catalytic efficiency (*k*_cat_/*K*_m_) of the PQS dioxygenase AqdC^II^ was found to be about 1.7-fold higher than that of AqdC^I^. Strain P13 and the majority of the isolates harboring the *aqd* gene cluster produce the AqdC^II^ variant. Considering that PQS levels in *P. aeruginosa* cultures are about 16 and 2 μM when cultivated in LB and artificial sputum medium, respectively (Collier et al., 2002; Lépine et al., 2003), and those in CF sputum can reach high nM ranges (Barr et al., 2015), the AqdC^II^ protein with its lower *K*_m_ value should perform better under physiological conditions.

Co-cultivation of *M. abscessus* isolates P13 and P5a with *P. aeruginosa* PAO1 reduced PQS concentrations in these cultures, whereas the presence of strain P4a (which lacks the *aqd* genes), or presence of dead mycobacterial cells significantly enhanced PQS production by *P. aeruginosa* PAO1. Thus, *P. aeruginosa* seems to recognize and respond to the presence of *M. abscessus* cells, cellular components, or exoproducts. Upregulation of PQS production by a mycobacterial effector should have broad implications on virulence as well as competitiveness of *P. aeruginosa*, because the *pqs* system controls a diverse array of virulence factors, such as the redox-active pigment pyocyanin, rhamnolipid surfactants, the siderophore pyoverdine, and the antimicrobial 2-alkyl-4-hydroxyquinoline *N*-oxides (Heeb et al., 2011). Especially the latter compounds not only affect competing microorganisms but also the fitness of *P. aeruginos*a itself, even promoting cell autolysis and DNA release (Hazan et al., 2016). As regards a possible mycobacterial effector, it is interesting that *P. aeruginosa* has been reported to enhance production of PQS and phenazine antimicrobials in response to *N*-acetylglucosamine, a peptidoglycan turnover product shed by Gram-positive bacteria (Korgaonkar and Whiteley, 2011; Korgaonkar et al., 2013). However, since mycobacteria have a complex cell envelope dominated by arabinogalactan and mycolic acids besides peptidoglycan, *P. aeruginosa* may sense additional or other components.

Remarkably, among the *M. abscessus* isolates tested, the presence of *aqd* genes strictly correlated with the subspecies *abscessus*, which is the subspecies most frequently isolated from CF patients worldwide (Bryant et al., 2016). It will be interesting to analyze whether the ability to rapidly inactivate *P. aeruginosa* QS signals, which not only control the production of virulence factors and antimicrobials but act as antimicrobials themselves, contributes to co-colonization competitiveness of the subspecies *abscessus.* However, AQ degradation by *M. abscessus* likely is only one aspect in a multi-faceted interaction between the two pathogens and our observation of increased PQS production in response to mycobacterial cell material opens up new questions of how *P. aeruginosa* monitors and responds to its biotic environment.

## Author Contributions

SF and FSB conceived the experiments. TK performed genome sequencing, TW performed and analyzed RT-qPCRs, and FSB performed all other experiments. JK analyzed genome sequences. KR and FB provided mycobacterial strains. SF and FSB analyzed data and wrote the paper. All authors contributed to the final version of the manuscript.

## Conflict of Interest Statement

The authors declare that the research was conducted in the absence of any commercial or financial relationships that could be construed as a potential conflict of interest.
