# Supplementary material for: Mycobacterium abscessus subsp. abscessus Is Capable of Degrading Pseudomonas aeruginosa Quinolone Signals
Source: Front Microbiol. 2017 Mar 2;8:339. doi: 10.3389/fmicb.2017.00339 (PMC5332414; doi:10.3389/fmicb.2017.00339)
Supplement: Supplementary file 1 [file Image_1.PDF]

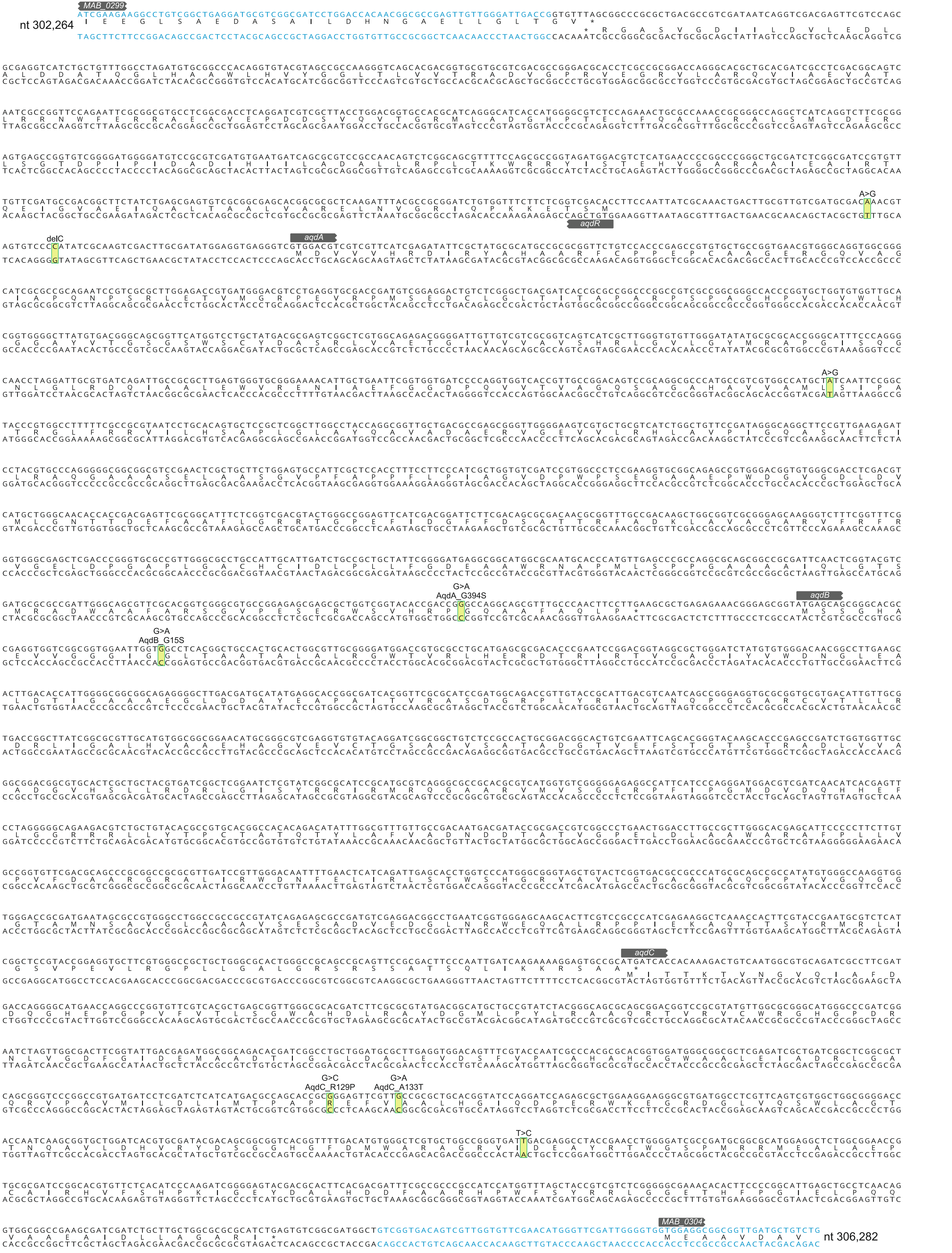

Supplementary Figure 1. Translated nucleotide sequence of the *aqdR-aqdABC* region (nucleotide 302.264 to 306.282) in the completely sequenced genome of the *Mycobacterium abscessus* type strain ATCC 19977 (NCBI Reference Sequence: NC_010397.1). The flanking regions in *M. abscessus* genomes in which the *aqd* gene cluster is absent are indicated by blue font. Starts of protein-coding regions are denoted by black arrows. Mutations in different *M. abscessus* isolates are indicated by green boxes and the respective changes in nucleotide and amino acid sequences are described.
